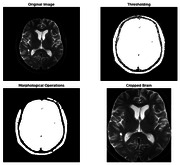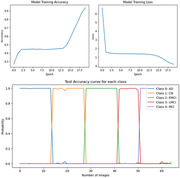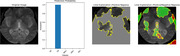# Neural Imaging for Alzheimer’s Prediction using AI: Exploring CNNs and LIME Explanations

**DOI:** 10.1002/alz.088802

**Published:** 2025-01-09

**Authors:** Abraham Varghese, Vinu Sherimon, Xavier C. Raja, Ben George Ephrem, Prasanth Gouda

**Affiliations:** ^1^ University of Technology and Applied Sciences, Muscat, Muscat Oman; ^2^ National University, Muscat, Muscat Oman

## Abstract

**Background:**

This study explores Alzheimer’s prediction through brain MRI images, utilizing Convolutional Neural Networks (CNNs) and Lime interpretability. Based on an extensive ADNI MRI dataset, we demonstrate promising results in predicting Alzheimer’s disease. Local Interpretable Model Agnostic Explanations (LIME) shed light on decision‐making processes, enhancing transparency. Using artificial intelligence to predict Alzheimer’s disease, this work emphasizes its importance in improving accuracy and interpretability, thereby advancing the field and improving diagnostics.

**Method:**

Alzheimer’s Disease Neuroimaging Initiative (ADNI) brain MRI images undergo rigorous preprocessing, which includes focusing on brain regions, thresholding and morphological operations to improve image quality. The CNN model was constructed and trained to identify the features of diverse classes of AD, CN, EMCI, LMCI, and MCI in the dataset (Figure 1). With two convolutional layers (25 and 75 filters), maximum pooling, flattening, and dense layers (500, 250, 100 units), the CNN model was trained on a variety of ADNI classes (AD, CN, EMCI, LMCI, MCI). With 375,168,555 trainable parameters, the model is evaluated on split datasets, emphasizing interpretability through LIME analysis for transparent decision insight in neural imaging analysis. This comprehensive methodology ensures robust Alzheimer’s prediction, transparency, and interpretability in neural imaging analysis.

**Result:**

The training procedure resulted in an impressive 98.75% training accuracy after 20 epochs. For the evaluation, 65 diverse images from various classes were used, and all images were accurately classified (Figure 2). Focusing specifically on the CN class data, LIME explanation was applied to identify and emphasize regions that significantly influenced the model’s predicted class. LIME delineates positive and negative regions in its output according to features that support or counter the predicted class (Figure 3). This thorough analysis contributes to a clearer understanding of prediction and promotes transparency in decision rationale.

**Conclusion:**

AI is helpful for predicting AD using MRI, providing accurate diagnostics and allowing for the interpretation of complex imaging data to identify subtle patterns indicative of the progression of AD. By interpreting the LIME representation, we can gain a deeper understanding of how the neural network makes its predictions, by identifying key regions in the brain image that influence the model’s predictions.